# Notes

**Published:** 1902-08

**Authors:** 


### NOTES.

Recently a peasant woman at Ostergaard, in Silesia, had occasion to leave her house to do some work outside. When she returned her eight-weeks-old baby, which had been lying on the floor, had disappeared. All the rooms in the house were searched, but to no purpose. Eventually, one of the neighbors discovered the child in the kennel of a huge St. Bernard dog, the animal affectionately licking the infant with its tongue. The St. Bernard, whose litter of puppies had been taken away, had carried the child into its kennel and was treating it with all the affection of a human being.

For some time the police of Ghent have been using trained dogs to aid them, and the plan has been attended with much success. The use of dogs has enabled the municipality to get along with fewer policemen than otherwise would be needed, and a dog costs but six cents a day to feed. The training of the animal is found far less troublesome than was supposed, but care has to be taken in selecting dogs with a suitable disposition. They are especially employed in searching dark, out-of-the-way places difficult to reach, where dangerous characters may be concealed. Their leaping powers in scaling walls are found of great use. They are trained to pursue persons taking to the water to escape and those who run away.

A drove of ten steers that were fattened on a farm in Providence township have been sold at nine cents per pound, the highest price ever paid in Eastern Pennsylvania for steers. The average weight of the steers was 1600 pounds each. The steers were bought ten months ago, when they weighed 900 pounds each, and the increase in weight is considered remarkable.

Trained cats are the latest fad of French society women. Fashion decrees that the animal must be "educated" entirely by its owner, and several of the best-known women in Parisian society are giving an hour a day to training their pets.

An order has been issued by the police department of Berlin providing for the shooting of all cats found at large in the city, and a fine for any owner of a cat who allows the animal to wander from his home.

A sanitary milk-supply company, with a capital of $50,000, has been formed to insure the people of Reading a healthful and clean milk-supply.

Cutaneous parasites and naphthalin: Ebmayer has found the use of naphthalin in cases of larvæ of the œstridæ most useful. The naphthalin is dissolved in warm alcohol and rubbed in with olive oil. A very small quantity suffices for the whole surface of a horse's skin. Vaseline and naphthalin, 1 to 10, is very useful with lice in cattle.

Dr. Joseph J. Smith, of Albany, Oregon, reports four cases of dropsy of the amnion during the month of February. Two of the colts are alive and doing well, and all of the mares made a good recovery. Creolin as an antiseptic uterine douche was used with the internal administration of stimulants.

The docking of horses has been placed on the Michigan criminal calendar. Moreover, parties bringing docked horses into the State are required to register them with the county clerk.

The veterinary periodical formerly published under the name of the *Michigan Veterinary Review*, and which for a time ceased publication, has reappeared under the name of the *International Veterinary Review*, and has as collaborators Drs. L. L. Conkey, W. A. McLean, W. E. A. Wyman, B. O. Johnson, P. E. Heseltine, and George Hare, all veterinarians. Its typographical appearance has been very much improved since its re-issue.

Prof. Otto Alex. Siedamgrotzky, one of the instructors in the Dresden Veterinary College, died at Wiesbaden, Germany, on June 20th. Dr. Siedamgrotzky was a very frequent contributor to foreign veterinary journalism for a great many years.

Elephant for farm work: James Cahill, of Virginia, is probably the only person in the United States who has in regular use upon his farm an elephant which is used for farm work. With the swaying beast hitched up to a plough he can turn more ground than any of his neighbors with a team of horses, and when it comes to hauling logs the elephant will walk away with ease with logs which the best teams of his neighbors cannot move. The elephant eats little more than a horse, and does many times the work of one, is gentle and docile and little trouble, and Mr. Cahill is more than pleased with his experiment. Mr. Cahill bought the elephant from a stranded circus proprietor.

The Zenner Disinfectant Co., of Detroit, Mich., are issuing a series of booklets containing the description of many of the prize animals of the most important fairs in the Western States. These descriptions are many of them by students and those connected with the agricultural colleges in the West, and are a source of information as to the best merits of the various types of animals. Copies of these booklets may be had by addressing the company at Detroit, Mich.

At Altoona, Penna., several very valuable thoroughbred Holstein cows were lost through eating dynamite which had rolled on the grass from a quarryman's shanty in the pasture field.

The passing out of existence of the old and well-known instrument firm of Messrs. John Reynders & Co., of New York, is an unexpected change. It is well for the profession's convenience that we are not to lose its advantage, inasmuch as Mr. Theodore F. Krey, who formerly managed this department, will continue this part of their business at 141 W. 54th Street, New York City.

Dr. Berg, Secretary of the National Museum of Buenos Ayres, died in January of this year. He was specially active in promoting the interests of this library.

